# Are New Place Representations Independent of Theta and Path Integration?

**DOI:** 10.1016/j.neuron.2014.05.011

**Published:** 2014-05-21

**Authors:** Robin Hayman, Neil Burgess

**Affiliations:** 1 UCL Institute of Cognitive Neuroscience, London, WC1N 3AR, UK; 2 UCL Institute of Neurology, Queen Square, London, WC1N 3BG, UK

## Abstract

Brandon et al. (2014) show that the formation of place cell representations in new environments is preserved under septal inactivation, and is thus likely independent of the hippocampal theta rhythm and, by implication, the firing of entorhinal grid cells and the process of path integration.

## Main Text

Place cells in the hippocampus of freely foraging rats, and other mammals including humans, appear to provide the neural basis for our sense of self-location (O’Keefe and Nadel, 1978). By providing one of the clearest links between neuronal firing and cognition, their discovery raised an important philosophical question, namely, is our sense of space constructed internally or is it derived from our sensory environment? Following Emmanuel Kant, O’Keefe and Nadel (1978) argued that the basic metric for space must be derived internally, from self-motion, onto which sensory experience could be associated. This position was fleshed out by McNaughton et al. (1996), who proposed that place cells form preconfigured continuous attractor networks, in which activity patterns are updated by self-motion or “charts.” In this view, environmental sensory information provides a secondary input: becoming associated with the “chart” active in a familiar environment so that it can be occasionally reset by environmental inputs to prevent the otherwise inevitable accumulation of error.

At around the same time as the charts idea took hold, the extent of environmental control over place cell firing was becoming clear: their firing locations maintaining fixed conjunctions of distances to environmental boundaries during parametric deformations of environmental size and shape (O’Keefe and Burgess, 1996). These findings suggest a feedforward model in which place cell firing is determined by environmental sensory inputs tuned to respond at specific distances from environmental boundaries in specific allocentric directions (“boundary vector cells” or “BVCs”; Hartley et al., 2000).

Beyond the obvious consensus that the spatial determinants of place cell firing comprise both environmental sensory inputs and path integration, controversy remains to this day regarding which input is primary: do environmental inputs become associated with a preconfigured path-integrating chart, or does path integration provide short-term stability in support of a primarily sensory environmental map?

In the intervening years, putative neural bases for both types of input have been found. Grid cells in medial entorhinal cortex (mEC; Hafting et al., 2005) are thought to support path integration, providing a metric for space based on self-motion that manifests similarly across environments (McNaughton et al., 2006). The regular arrangement of their firing fields across an environment, and the fixed offsets between the firing patterns of neighboring cells, suggest internal dynamics. Equally, putative BVCs have been found, whose firing is determined by the distance and direction of environmental boundaries across different environments, in subiculum (Lever et al., 2009) and rather similar “border cells” found in entorhinal cortex (Solstad et al., 2008). However, the controversy as to which might be the primary input to place cells has remained.

A similarly controversial question has concerned the role of the theta rhythm—is it an epiphenomenon of rate-coded neural processing, or does it play a functional role, and if so, what role does it play? The movement-related theta rhythm seen in freely moving rodents is a large-amplitude local field potential oscillation of 4–8 Hz, which strongly modulates the firing of place cells and a large proportion of grid cells. In support of a functional role for theta rhythmicity, the theta phase of firing of place cells and grid cells correlates with distance traveled through the firing field—providing information beyond that carried in the firing rate alone (see Burgess and O’Keefe, 2011 for a review). Thus, theta rhythmicity might contribute to path integration by allowing firing phase to integrate movement to calculate displacement. In this view, theta rhythmicity is thought to underlie the mechanism by which grid cell firing supports path integration, in contrast to environmental inputs such as boundary vector cells, see e.g., Burgess and O’Keefe (2011). However, reports of place cell and grid cell firing in the absence of theta rhythmicity in crawling bats have argued against any important functional role for the theta rhythm.

Two previous experiments examined the role of theta rhythmicity in grid cell firing in rodents by inactivating the septum, which severely disrupts the hippocampal theta rhythm (Brandon et al., 2011; Koenig et al., 2011). They found that the extent of disruption of theta was specifically predictive of the disruption of grid cell firing, with weaker effects on the firing of other spatial cells such as head-direction cells, place cells, and nongrid spatial cells, including examples of boundary vector cells (Koenig et al., 2011). These results suggested that theta rhythmicity does play a role in grid cell firing in rodents and also that place cell firing can be somewhat independent of grid cell firing. This latter finding is consistent with the developmental time course, from which it has been argued that place cell firing could not be driven by grid cell firing, because stable place cell firing precedes stable grid cell firing (Wills et al., 2010), although stable boundary-related firing is seen at this early developmental stage (Bjerknes et al., 2014).

However, from the “charts” point of view, grid cell-mediated path integration could determine the initial place cell representation in a new environment; environmental sensory associations then stabilize place cell firing as the environment becomes familiar and could replace the original grid cell input.

To test the charts hypothesis, Brandon et al. (2014) recorded place cell firing in novel and familiar environments while disrupting hippocampal theta by inactivating the septum. They found, as before, a severe reduction in theta power in the LFP in hippocampus and mEC and in the theta rhythmicity of place cell firing. This level of reduction corresponded to complete disruption of grid cell firing patterns in a previous paper using muscimol inactivation (Brandon et al., 2011) and in two grid cells recorded in the current study. There was also little effect of the septal inactivation on place cell firing in the familiar environment (apart from a slight reduction in the size of firing fields).

When the rats were put into a novel environment, normal levels of place cell “remapping” were seen (i.e., generation of new, orthogonal, firing patterns in the new environment compared to the familiar one). The new firing patterns were unchanged by recovery from the inactivation 24 hr later. Thus, the formation of new place cell representations in a novel environment appears not to require theta rhythmicity or grid cell firing patterns. This contradicts suggestions that the spatial modulation of place cell firing reflects mechanisms dependent on theta oscillations (see Burgess and O’Keefe, 2011 for a review). If it is true that grid cells implement a preconfigured metric based on path integration or “chart” (McNaughton et al., 2006), then this result also suggests that new place cell representations are not built on such charts.

Nonetheless, a slight reduction in place cell firing rates was observed in the inactivation group, and the characteristic increase in stability during the 30 min trial in control animals was reduced in the inactivation group. This suggests that grid cells do have a functional input to place cell firing and that this input strengthens with experience of a new environment and improves the spatial stability of place cell firing, even if it does not determine their firing fields.

This study raises several interesting questions, aside from the debate about the primacy of sensory input versus path integration. The septum provides an important cholinergic input to the hippocampus in addition to driving theta rhythmicity, and both inputs are inactivated in the current study. Future experiments, using, for example, cell-type-specific optogenetic manipulations will be able to dissociate the contributions of these two inputs. Indeed, cholinergic input is thought to play an important role in novelty processing by place cells. However, Brandon et al. (2014)’s findings indicate that neither the septal cholinergic input nor the theta rhythmicity are required for the formation of novel place cell representations.

The relationship between place cell remapping and grid cell firing has been the subject of much study. For example, shifts in the spatial firing patterns of different modules of grid cells relative to each other (Stensola et al., 2012) might drive the remapping of place cells. However, the reverse relationship is also possible, that place cells anchor the grid firing patterns to the environment (e.g., Burgess and O’Keefe, 2011), or place cell remapping might be independent of grid shifts (which occur during environmental manipulations that do not typically cause place cell remapping, cf. O’Keefe and Burgess, 1996; Stensola et al., 2012).

Finally, it is important to note the presence of cells in the mEC encoding direction. These cells appear to be fully present at the earliest developmental stage at which rat pups begin to move from the nest and are thought to contribute to both types of input to place cells. They are required for encoding environmental boundaries (for which the BVCs need directional tuning as well as distance tuning) and for path integration, for which representations need to be updated in terms of movement direction, whether via theta-related mechanisms or within a continuous attractor chart (albeit that these cells encode head direction rather than movement direction). As with the locational tuning of place cells, the directional tuning of these cells also shows a combination of environmental input and updating by self-motion, and rotation of their directional tuning goes hand-in-hand with rotation of the orientation of place and grid representations.

In summary, Brandon et al. (2014)’s results shed light on how the place cell representation of space is built and bring the focus back onto sensory environmental inputs, such as boundary vector cells, with a supporting role for theta rhythmicity and grid cell firing patterns, which have been associated with spatial representation based on path integration.
